# A New Low-Temperature Solder Assembly Technique to Replace Eutectic Sn-Bi Solder Assembly

**DOI:** 10.3390/mi13060867

**Published:** 2022-05-31

**Authors:** Lingyao Sun, Zhenhua Guo, Xiuchen Zhao, Ying Liu, Kingning Tu, Yingxia Liu

**Affiliations:** 1 School of Materials Science and Engineering, Beijing Institute of Technology, Beijing 100081, China; sunlingyao_513@163.com (L.S.); guozhenhua1318@163.com (Z.G.); zhaoxiuchen@bit.edu.cn (X.Z.); yingliu@bit.edu.cn (Y.L.); 2 Department of Materials Science and Engineering, City University of Hong Kong, Hong Kong, China; kntu@cityu.edu.hk; 3 Department of Electrical Engineering, City University of Hong Kong, Hong Kong, China; 4 Department of Advanced Design and System Engineering, City University of Hong Kong, Hong Kong, China

**Keywords:** low-temperature soldering, 3D IC, Bi aggregation, Sn-Bi solder

## Abstract

We successfully achieved low-temperature assembly by reflowing the 13.5Sn-37.5Bi-45In-4Pb quaternary eutectic solder paste and the SAC 305 solder ball together at 140 °C for 5 min. The wetting angle of the mixed solder joint is 17.55°. The overall atomic percent of Pb in the mixed solder joint is less than 1%, which can be further reduced or eliminated. Moreover, after aging at 80 °C for 25 days, we observed no obvious decrease in shear strength of the fully mixed solder joint, which is the most advantage of this assembly technique over Sn58Bi solder assembly. The Bi phase segregation at the interface is slowed down compared with Sn-Bi solder joint. This low-temperature assembly is promising to be applied in advanced packaging technology to replace the eutectic Sn-Bi solder.

## 1. Introduction

As the downscaling trend of silicon chips is approaching its physical limits, advanced packaging technologies that integrate multiple chips, either vertically or horizontally, provide an alternative approach for developing post-Moore-era electronics [1,2,3]. The vertical or horizontal stacking of chips in advanced packaging technology requires a low-melting-point solder. Specifically, vertical stacking such as 3D integrated circuit (3D IC) is achieved via multiple reflows, and the use of a low-melting-point solder can prevent the re-melting of solder joints connected in a previous reflow [4]. In addition, packaging sizes generated by horizontal stacking are becoming increasingly large, leading to severe warpage problems [5]. This warpage can be relieved by using a low-melting-point solder during assembly. Thus, low-temperature assembly enables vertical and horizontal advanced packaging to be achieved, supporting continuing advances in microelectronic devices.

Currently, the packaging industry is trying to find an appropriate low-melting-point solder alloy. Tin–bismuth (Sn–Bi) eutectic solder has a melting point of 139 °C, but its brittleness limits its application in mobile electronic devices, especially after aging [6,7,8,9,10,11,12]. On the one hand, this is because aging causes Bi atom to segregate at the interface between the solder joint and the substrate [11,12]. As Bi is inherently brittle, this segregation increases the likelihood of interfacial fracture during drop testing. On the other hand, after aging, the IMC at the interface between the solder joint and the substrate will grow and thicken with the aging time. Due to the brittleness of the IMC, excessive thickness will reduce the mechanical properties and reliability of the joint [13,14,15,16,17]. In addition, the wettability of solder is also an important evaluation in electronic packaging industry. Since Bi will reduce the reaction speed of Sn and Cu, the wetting time will increase and the wettability will decrease [18]. Sn-Bi solder has very limited utility in mobile devices due to its brittleness and poor wettability. Tin–indium (Sn-In) eutectic solder has a suitable melting point (118 °C), and bonds well with Cu, Ni, and Au substrates [19]. However, Sn-In solder is too soft, in addition, the widespread use of low-temperature solder with high In content is economically unfavorable because In is very expensive [20]. Most studies on low-melting-point solders have explored whether the addition of minor amounts of a third or fourth element into eutectic Sn-Bi alloys can produce alloys with better mechanical properties. These studies have not been successful, as the brittle nature of eutectic Sn–Bi has proven to be largely unmodifiable [21]. Additionally, there have been attempts to obtain a composite solder with better performance by mixing the two solders [22,23,24,25,26,27]; however, the reflow temperature is still high. Thus, there is an urgent need for a low-temperature assembly technology to replace eutectic Sn-Bi for use in industrial applications of mobile devices. In this paper, we report a low-temperature assembly technology to do so. Moreover, the assembled solder joint has a relatively stable shear test performance even after a long time of aging.

## 2. Experimental Section

**Figure 1:**
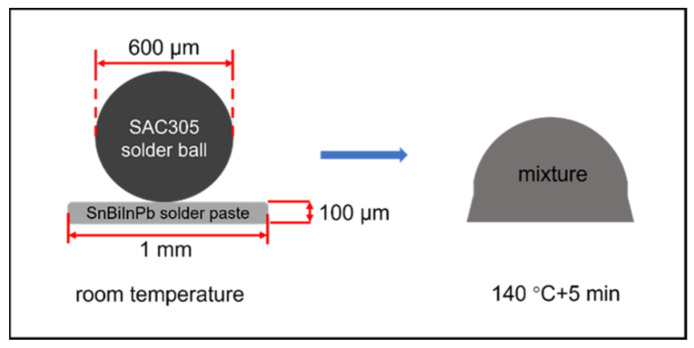
Schematic diagram of the assembly process using SAC305 solder balls and 13.5Sn-37.5Bi-45In-4Pb solder paste.

**Figure 2:**
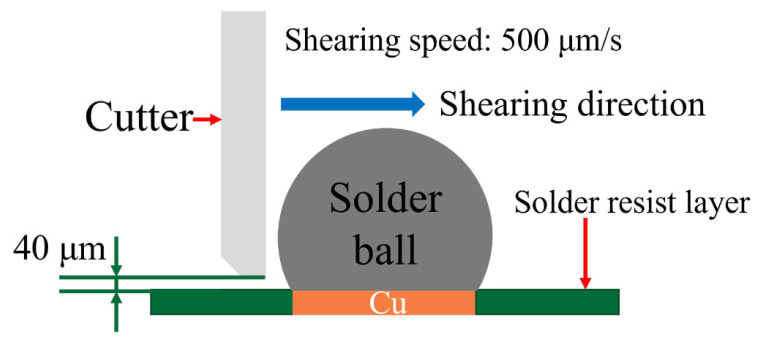
Schematic diagram of solder joint shear strength test.

The prepared Cu solder pads were ultrasonically cleaned with acetone, dilute hydrochloric acid, deionized water, and alcohol to remove surface oil and oxides. The Cu pads we used are 1 mm in diameter. And we developed a solder paste of 13.5Sn-37.5Bi-45In-4Pb quaternary eutectic alloy, which has a melting point of 60 °C. We use a low-temperature flux in the preparation of the 13.5Sn-37.5Bi-45In-4Pb solder paste, which is developed by the Beijing Institute of Nonferrous Metals and Rare Earth Applications. It works well in the range of 50~150 °C. The method of preparing the solder paste will be presented in our following paper. We printed the solder paste on the Cu pads with a designed stencil. A Sn96.5Ag3.0Cu0.5 (SAC305) solder ball was placed on each pad. The printed quaternary eutectic ultra-low-temperature solder paste is a cylinder of 1 mm in diameter and 100 μm in height. The SAC305 solder ball is 600 μm in diameter, as shown in Figure 1. Then, the substrate was reflowed at 100 °C, 120 °C, 140 °C, and 160 °C for 5 min. The reflowing temperature is higher than the melting point of 13.5Sn-37.5Bi-45In-4Pb solder paste and lower than the melting point of SAC305 solder balls. During reflow, the quaternary eutectic solder paste melts into a liquid state, while the SAC305 solder balls remain solid, which then gradually dissolve into the liquid solder. Figure 2 is a schematic diagram showing our low-temperature assembly strategy. After the reflow, we mounted and polished the samples to obtain cross-sectional images, which were observed by scanning electron microscope (SEM, Regulus 8230). The phase composition of the mixed solder before and after aging was determined by the energy dispersive spectrometer (EDS) and X-ray Diffractometer (XRD). The density of 13.5Sn-37.5Bi-45In-4Pb quaternary eutectic solder paste was measured by the Archimedes drainage method.

The shear test was carried out on a PTR-1100 shear testing machine. The shear speed is 500 µm/s with a height of 40 µm, which is shown schematically in Figure 2. Additionally, 10 samples under each reflow condition were tested. The mixed solder bumps, obtained after reflowing at 140 °C for 5 min, were aged at 80 °C for 5 days, 15 days, and 25 days, respectively. Then, the microstructure of the aged solder joint was observed by SEM, and its phase composition was analyzed by EDS. We also tested the maximum shear strength of the fully mixed solder joint after aging by PTR-1100 shear testing machine.

## 3. Results and Discussion

### 3.1. Reflow Results

**Figure 3:**
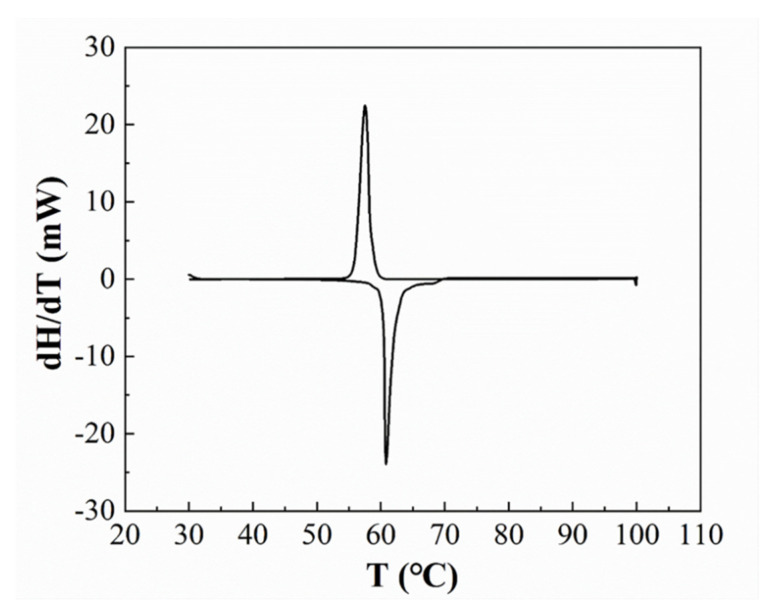
The DSC curve of 13.5Sn-37.5Bi-45In-4Pb solder paste.

**Figure 4:**
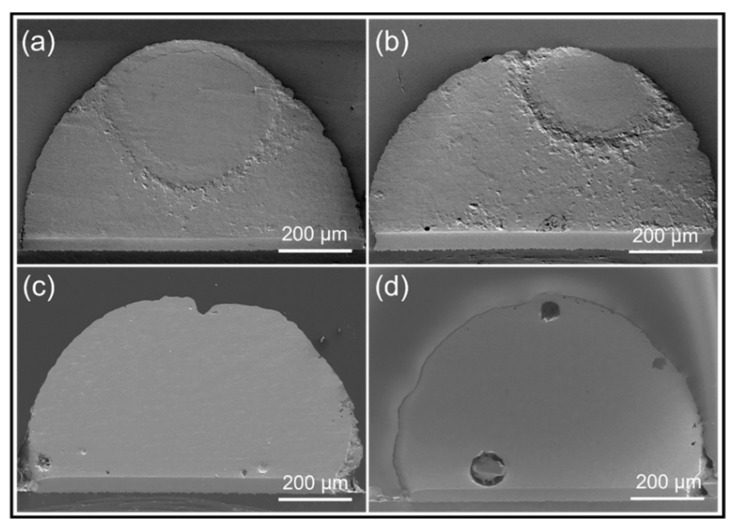
Overall SEM images of 13.5Sn-37.5Bi-45In-4Pb solder paste and SAC305 solder ball after reflowing at (**a**) 100 °C, (**b**) 120 °C, (**c**) 140 °C and (**d**) 160 °C for 5 min, respectively.

We choose a reflow temperature that is between the melting point of SAC305 (at 230 °C) and the melting point of 13.5Sn-37.5Bi-45In-4Pb solder paste (at 60 °C). The DSC curve of 13.5Sn-37.5Bi-45In-4Pb solder paste is shown in Figure 3. Therefore, solid–liquid inter-diffusion happens during the assembly process. During the solid–liquid inter-diffusion, Sn atoms from SAC305 solder ball will dissolve into the molten 13.5Sn-37.5Bi-45In-4Pb solder paste, and the two parts are mixed together. The fully mixed solder joint obtained after reflowing at 100 °C, 120 °C, 140 °C, and 160 °C for 5 min are shown in Figure 4. According to Figure 4a,b, there is still a clear boundary between the two parts after reflowing at 100 °C and 120 °C for 5 min, but a completely mixed and uniform solder bump is achieved after reflowing at 140 °C and 160 °C for 5 min.

**Figure 5:**
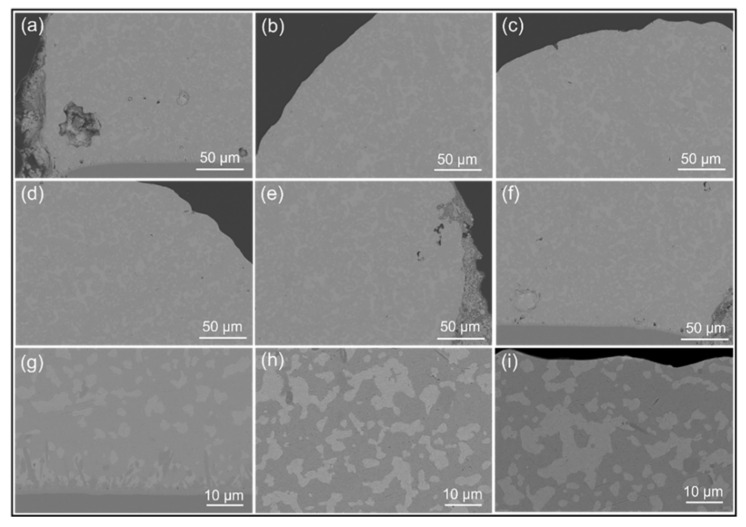
The fully mixed solder joint obtained after reflowing of 13.5Sn-37.5Bi-45In-4Pb solder and SAC305 at 140 °C for 5 min; (**a**–**f**): the 500× magnification of the lower left, left, upper-left edge, upper-right edge, right and lower right parts of the solder joint; (**g**–**i**): the 2000× enlarged view of the connection between the bottom of the solder joint and the Cu plate, the middle and top of the solder joint.

Higher magnification SEM images in Figure 5 show the uniformity of the microstructure after reflowing at 140 °C for 5 min. Figure 5a–f are the 500× magnification SEM images of the lower left, left, upper-left edge, upper right-edge, right, and lower right parts of the solder joint, respectively, and (g–i) are the 2000× enlarged view of the connection between the solder joint and the Cu plate, the middle and top of the solder joint. According to Figure 5, the composition of the mixed solder obtained after reflowing at 140 °C for 5 min is completely homogeneous. Therefore, 140 °C is considered the optimum temperature to obtain a fully mixed solder of 13.5Sn-37.5Bi-45In-4Pb and SAC305.

### 3.2. Composition of the Fully Mixed Solder Joint

We calculated the composition of the fully mixed solder joint with the value of volume and density. The volume of the solder paste and solder ball is calculated from the dimensions marked in Figure 1. The density of SAC305 solder balls is 7.37 g/cm^3^ provided by the manufacturer, and the density of 13.5Sn-37.5Bi-45In-4Pb quaternary eutectic solder paste is 8.01 g/cm^3^ tested by Archimedes drainage method. Then, the atomic percentage of each element is calculated and listed in Table 1.

**Figure 6:**
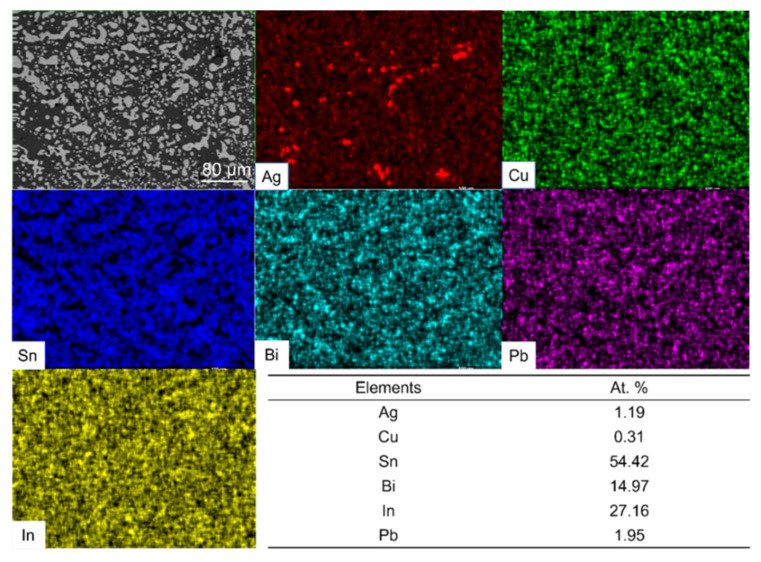
Elemental distribution and atomic percentage results of fully mixed composite solder joint.

We performed the EDS mapping of the fully mixed solder joint after reflowing at 140 °C for 5 min, and the results are shown in Figure 6. Comparing the atomic percentage table in Figure 6 with Table 1, the difference in the content of each element is not significant and within the measurement error.

From Figure 5 and Figure 6, we can conclude that a well-mixed solder joint can be achieved by the 140 °C reflow of SAC305 solder ball and 13.5Sn-37.5Bi-45In-4Pb solder paste for 5 min. One thing that needs to be noted is the overall Pb atomic concentration in the mixed solder is about 1%. With further optimization, we can reduce the Pb atomic concentration to less than 0.5%. We can reduce the relative Pb content in the fully mixed solder by further reducing the amount of 13.5Sn-37.5Bi-45In-4Pb eutectic solder paste or increasing the amount of SAC305 solder. Specifically, this can be achieved by using thinner stencils (such as 50 μm, 30 μm) or by using larger diameter SAC305 solder ball. We will also try to reduce or even eliminate the Pb used in the low-melting-point solder paste. This means that the technology is promising to be applied to consumer products.

**Figure 7:**
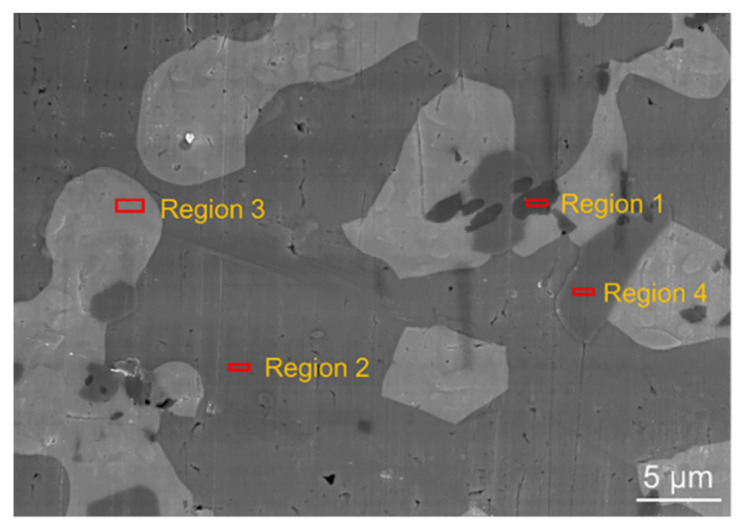
Local magnification SEM image of the fully mixed solder joint.

**Figure 8:**
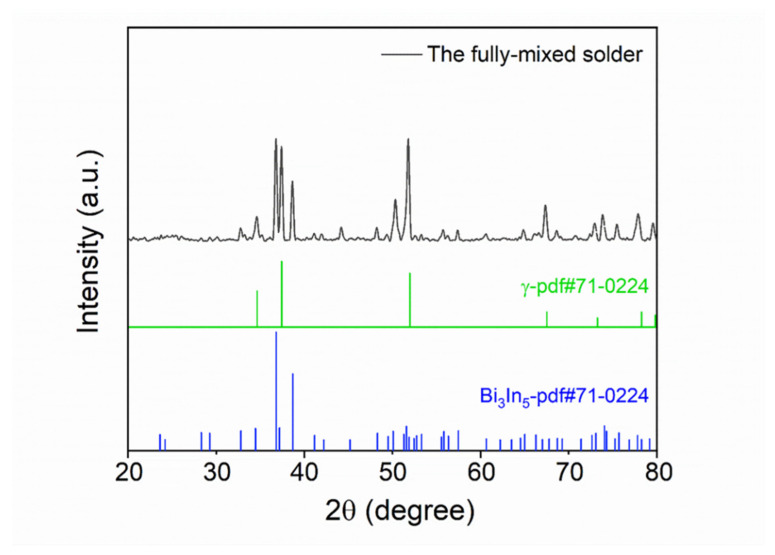
XRD analysis results of the mixed solder joint formed by SAC305 and 13.5Sn-37.5Bi-45In-4Pb after reflowing at 140 °C for 5 minutes.

**Figure 9:**
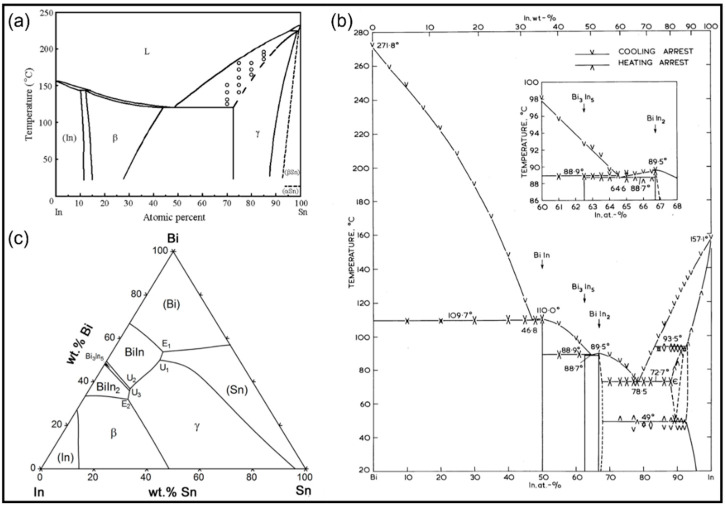
(**a**) The Sn-In phase diagram [28]; (**b**) the Bi-In phase diagram [29]; (**c**) the Bi-In-Sn phase diagram [30].

We further investigated the microstructure of the mixed solder joint after reflowing at 140 °C for 5 min with EDS and XRD, as shown in Figure 7 and Figure 8 and Table 2. According to Figure 7 and Table 2, there are four phases in the mixed solder joint, including the Cu_6_Sn_5_, γ-phase, Bi_3_In_5_, and Ag_3_Sn phases. The Cu_6_Sn_5_ phase is generated by the interfacial reaction between the solder joint and the Cu substrate, which is illustrated in the darkest area marked as region 1 in Figure 7. The γ-phase is a Sn-rich phase similar to the one marked in the Sn-In phase diagram shown in Figure 9a [28]. Bi_3_In_5_ is an intermetallic compound phase formed in the reaction between Bi and In [29,30], and so does Ag_3_Sn, formed in the reaction between Ag and Sn [31]. Bi-In and Bi-Sn-In phase diagrams are shown in Figure 9b,c. All four phases are not pure and have substitutions from other elements. The XRD results in Figure 8 show that the two main phases in the mixed solder joint, Bi_3_In_5_ and γ-phase, are consistent with the EDS results [30]. The amount of Cu_6_Sn_5_ and Ag_3_Sn phases is too little to be observed in the XRD results.

### 3.3. Wettability

**Figure 10:**
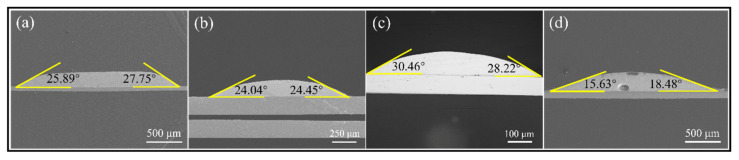
Wetting angle: (**a**) 13.5Sn-37.5Bi-45In-4Pb quaternary eutectic solder paste; (**b**) SAC305 solder ball; (**c**) Sn58Bi solder paste; (**d**) the fully mixed solder of 13.5Sn-37.5Bi-45In-4Pb quaternary eutectic solder paste and SAC305 solder ball.

To evaluate the wettability of composite solder joint where SAC305 solder ball is completely mixed with 13.5Sn-37.5Bi-45In-4Pb quaternary eutectic solder paste, we reflow the mixed solder directly on the cleaned Cu substrate. The wetting angle was measured, and that of SAC305 solder ball, Sn58Bi solder paste, and 13.5Sn-37.5Bi-45In-4Pb quaternary eutectic solder paste also were tested, respectively. The results are shown in Figure 10, where the four were compared. The wetting angle of the mixed solder is only 17.05°, while the wetting angles of the 13.5Sn-37.5Bi-45In-4Pb quaternary eutectic solder paste, SAC305 solder ball, and Sn58Bi solder paste are 26.82°, 24.24°, and 29.34°, respectively. Therefore, the wettability of our mixed solder is the most excellent among the four.

### 3.4. Aging Performance of the Mixed Solder Joint

**Figure 11:**
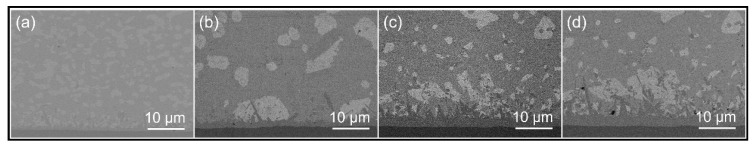
SEM images of the interface between the fully mixed solder joint and the Cu substrate: (**a**) before aging, (**b**) 5 days, (**c**) 15 days, and (**d**) 25 days of aging.

**Figure 12:**
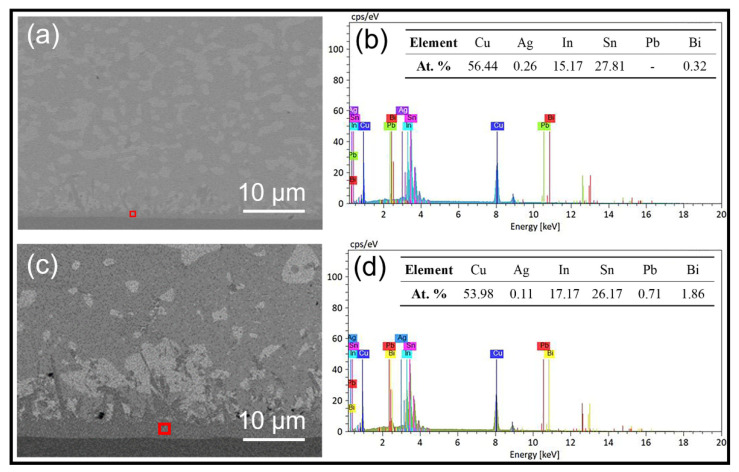
Composition analysis of IMC: (**a**) before aging and (**c**) after 25 days of aging: (**b**) EDS results of the region marked in (**a**); (**d**) EDS results of the region marked in (**c**).

The solder bumps obtained after reflowing at 140 °C for 5 min were thermally aged at 80 °C for 5 days, 15 days, and 25 days [32]; the results are shown in Figure 11a–d. The thicknesses of intermetallic compounds (IMC) generated by the reaction between the mixed solder and the interface before aging and after aging for 5, 15, and 25 days were measured as 1.06 μm, 2.39 μm, 3.66 μm, and 4.08 μm, respectively, as shown in Table 3. The composition analysis of IMC before aging and after aging for 25 days was carried out using EDS, as shown in Figure 12. It was found that the IMC was Cu_6_(Sn, In)_5_, with In substitute some Sn atoms in Cu_6_Sn_5_ IMC. 

After aging for 15 and 25 days, we observe the coarsening effect of Bi_3_In_5_ phase. We also observe slight aggregation of Bi_3_In_5_ phase at the interface. The aggregated phase of Bi_3_In_5_ is not continuous, which may be due to the spinous shape of intermetallic compound at the solder and Cu interface.

**Figure 13:**
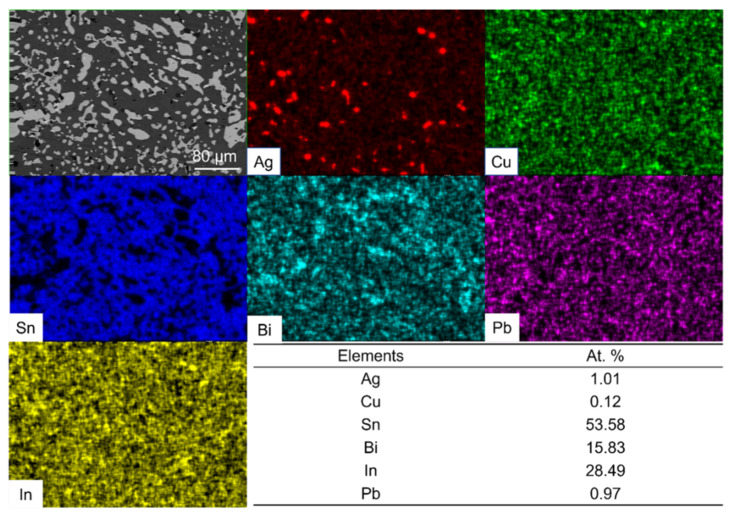
Elemental distribution and atomic percentage results of fully mixed solder joint after 25 days of aging.

Figure 13 shows the EDS Mapping of the mixed solder joint after 25 days of aging. In Figure 13, the element distribution is uniform throughout the solder joint. Further comparison of the tables in Figure 6 and Figure 13 shows that there are also no significant changes in the atomic percentage of the elements.

**Figure 14:**
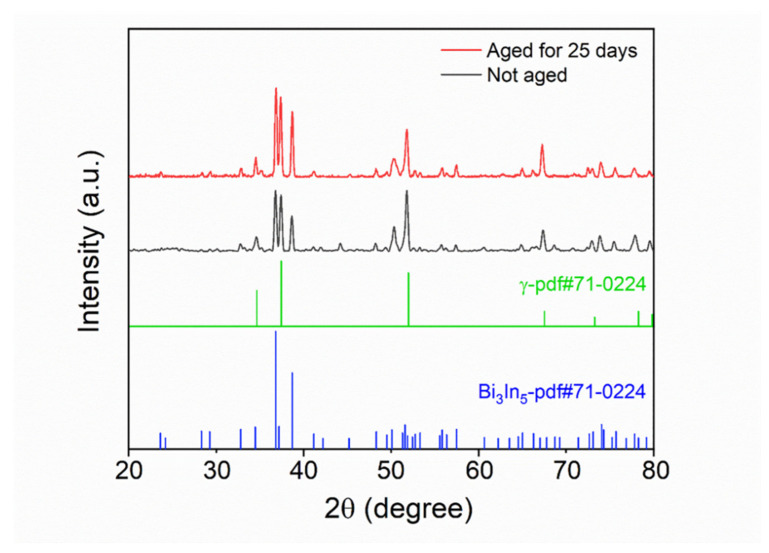
XRD analysis results of the mixed solder joint before and after aging for 25 days.

We put the results of XRD analysis before and after aging together for comparison, as shown in Figure 14. The main components of the phases before aging and after aging remain the same, which are the γ-phase and Bi_3_In_5_ phase. Figure 13 and Figure 14 demonstrate that the microstructure of the mixed solder joint is stable after aging.

### 3.5. Shear Test of the Fully Mixed Solder Joint

**Figure 15:**
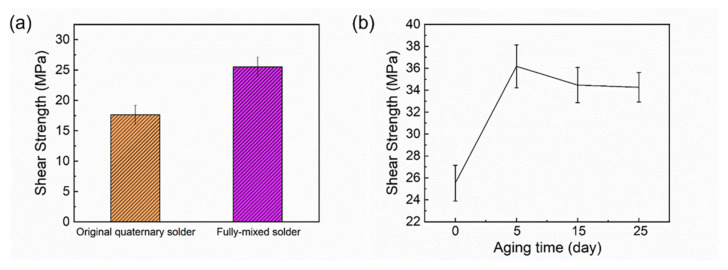
(**a**) Shear strength of the original quaternary solder joint and the fully mixed solder joint; (**b**) Variation of shear strength of fully mixed solder joint with aging time.

The shear strength of the mixed solder joint was tested and compared with that of the original 13.5Sn-37.5Bi-45In-4Pb quaternary solder formed after reflowing at 140 °C for 5 min, as shown in Figure 15a. The shear strength of the original 13.5Sn-37.5Bi-45In-4Pb quaternary solder joint is only 17.62 (±1.57) MPa, while the mixed solder joint is 25.53 (±1.63) MPa, which was much higher than that of the original quaternary solder joint. In addition, the shear strength of SAC305 is 23 MPa [33], so the shear strength of the mixed solder is similar to SAC305.

**Figure 16:**
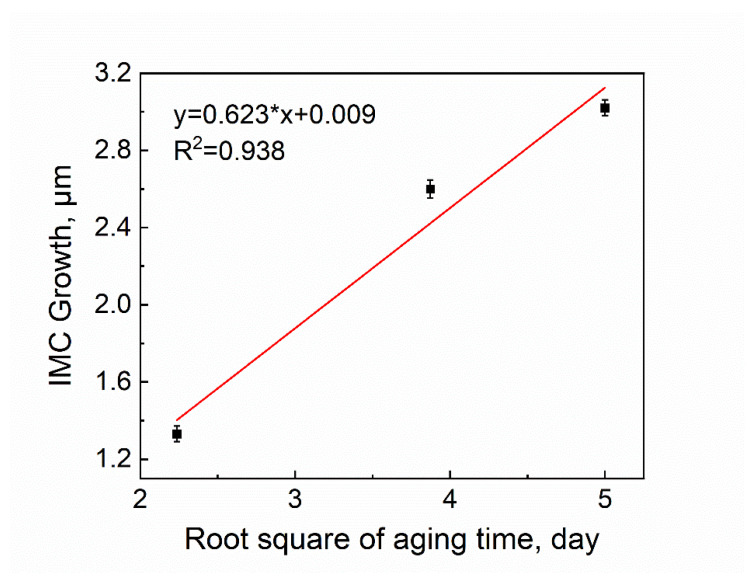
Linear relationship between the thickness of total IMC and the square root of aging days in the fully mixed solder joint.

The shear strength of the samples after aging for 5, 15, and 25 days after reflowing at 140 °C for 5 min is shown in Figure 15b. The shear strength of the samples before aging was 25.53 (±1.63) MPa, and the shear strength of the samples after aging at 80 °C for 5, 15, and 25 days were 36.18 (±1.96) MPa, 34.47 (±1.61) MPa, and 34.27 (±1.34) MPa, respectively. After 5 days of aging, the shear strength is increased and there is almost no decrease in shear strength with increasing aging time. Because the thickness of the IMC has an important effect on the performance of the solder joint, the IMC needs to be thick enough to ensure a strong and reliable bonding. However, due to the brittleness of the IMC, if it is too thick it will also reduce the joint reliability [14,15,17]. After 5 days of aging, the shear strength increased due to the growth of IMC. Table 3 shows that although the IMC thickens after aging, it is not over-thickened, resulting in good shear strength. It has been shown that the thickness of IMC at the Sn58Bi/Cu interface increases linearly with the square root of the aging time [34,35,36,37], that is, the IMC growth at the interface in solder joint follows the empirical diffusion formula:

(1) $$X={\left(Dt\right)}^{\frac{1}{2}}+X_{0}$$

where X is the total IMC thickness, $X_{0}$ is the initial IMC thickness, t is the aging time, and D is the diffusivity of the IMC layer [37]. The growth of IMC thickness $\left(X-X_{0}\right)$ is plotted with the square root of aging days $\left(t^{\frac{1}{2}}\right)$, and the results are shown in Figure 16. The growth of IMC with aging time for Sn58Bi was compared with published results [37]. The IMC growth rate of Sn58Bi aged at 135 °C was reported to be 3.0 µm/day^1/2^, while the IMC growth rate of the fully mixed solder was only 0.623 µm/day^1/2^, which is much less than that of Sn58Bi.

The Bi containing low melting temperature solder, which can keep a stable shear strength after aging, is an important finding in our work. Many works have reported the shear strength decrease in Bi containing solder after aging [32,38,39], which is due to the large aggregation of Bi at the interface between substrate and solder [12]. A continuous decrease in the shear strength was reported with an increase in aging time of 7 days, 14 days, and 21 days at an aging temperature of 100 °C. The total decrease of shear strength can be 30~40% after aging for 21 days [8]. In our work, we show a much better shear test performance after aging. We tend to believe it is because the Bi atoms are stabilized by Bi_3_In_5_ phase in our solder joint. Bi_3_In_5_ phase is an intermetallic compound phase, and the diffusion of this phase is significantly slower than the diffusion of Bi atoms. Therefore, Bi atoms aggregation slows down, thus a better shear strength can remain and the brittleness of the solder joint can be mitigated after aging. The 140 °C assembly technique brought up in this letter can provide a similar reflow condition to eutectic Sn-Bi solder, while the mechanical performance after aging is much better than Sn-Bi solder. Therefore, the technique is promising to replace the eutectic Sn-Bi solder in advanced electronic packaging.

## 4. Conclusions

Low-temperature assembly is achieved by reflowing the SAC305 solder ball and 13.5Sn-37.5Bi-45In-4Pb solder paste together at 140 °C for 5 min. The fully mixed solder joint has a uniform microstructure, with two main phases of Bi_3_In_5_ and γ-phase, and it has very excellent wettability. After 5, 15, and 25 days of aging, the fully mixed solder joint shows a stable microstructure, and there is no continuous aggregation of Bi atoms at the interface between the solder joint and the Cu substrate. More importantly, there is almost no decrease in shear strength after aging for 25 days. The reason is explained as the stabilization effect of Bi atoms by Bi_3_In_5_ phase, and the IMC did not grow too thick after aging. This technology is promising to replace eutectic Sn-Bi solder in the electronic packaging industry, with a further study on the method of eliminating Pb. 

## Figures and Tables

**Table 1:** Calculation of the atomic percentage of each element in the fully mixed solder.

Elements	at. %
Ag	2.02
Cu	0.56
Sn	65.12
Bi	9.81
In	21.43
Pb	1.06

**Table 2:** Element content of the regions marked in Figure 7.

Element	Sn	In	Bi	Pb	Ag	Cu	Phase
Atom (%)							
Region							
1	43.23	1.13	1.57	0.24	0.19	53.64	Cu_6_Sn_5_
2	78.02	17.23	3.82	0.58	0.35	-	γ
3	3.07	60.12	36.20	0.07	0.31	0.23	Bi_3_In_5_
4	23.84	9.63	0.39	0.28	65.86	-	Ag_3_Sn

**Table 3:** The thickness of IMC of the fully mixed solder joints before aging and after aging for 5 days, 15 days, and 25 days.

Aging Time	The Average Thickness of IMC Layer (μm)
0 day	1.06
5 days	2.39
15 days	3.66
25 days	4.08
